# Pilot longitudinal mosquito surveillance study in the Danube Delta Biosphere Reserve and the first reports of *Anopheles algeriensis* Theobald, 1903 and *Aedes hungaricus* Mihályi, 1955 for Romania

**DOI:** 10.1186/s13071-016-1484-7

**Published:** 2016-04-11

**Authors:** Edina Török, Alexandru Tomazatos, Daniel Cadar, Cintia Horváth, Lujza Keresztes, Stephanie Jansen, Norbert Becker, Achim Kaiser, Octavian Popescu, Jonas Schmidt-Chanasit, Hanna Jöst, Renke Lühken

**Affiliations:** Department of Biology and Ecology, Babeş-Bolyai University, Cluj-Napoca, Romania; Romanian Academy Institute of Biology, Bucharest, Romania; Molecular Biology Center, Institute for Interdisciplinary Research in Bio-Nano-Sciences, Babes-Bolyai University, Cluj-Napoca, Romania; Bernhard Nocht Institute for Tropical Medicine, WHO Collaborating Centre for Arbovirus and Haemorrhagic Fever Reference and Research National Reference Centre for Tropical Infectious Diseases, Hamburg, Germany; German Mosquito Control Association (KABS), Institute for Dipterology, Speyer, Germany; University of Heidelberg, Heidelberg, Germany; Centre for Infection Research (DZIF), Partner site Hamburg-Luebeck-Borstel, Hamburg, Germany

**Keywords:** Romania, Danube Delta Biosphere Reserve, Mosquito surveillance, Mitochondrial cytochrome *c * oxidase subunit I, *Aedes hungaricus*, *Anopheles algeriensis*

## Abstract

**Background:**

Mosquito-borne viruses (moboviruses) are of growing importance in many countries of Europe. In Romania and especially in the Danube Delta Biosphere Reserve (DDBR), mosquito and mobovirus surveillance are not performed on a regular basis. However, this type of study is crucially needed to evaluate the risk of pathogen transmission, to understand the ecology of emerging moboviruses, or to plan vector control programmes.

**Methods:**

We initiated a longitudinal mosquito surveillance study with carbon dioxide-baited Heavy Duty Encephalitis Vector Survey traps at four sampling sites to analyse the spatio-temporal pattern of the (i) mosquito species composition and diversity, (ii) functional groups of mosquitoes (oviposition sites, overwintering stage, and number of generations), and (iii) the occurrence of potential West Nile virus (WNV) vectors.

**Results:**

During 2014, a total of 240,546 female mosquitoes were collected. All species were identified using morphological characteristics and further confirmed by mitochondrial cytochrome *c* oxidase subunit I (COI) gene analysis of selected specimens. The two most common taxa were *Coquilettidia richiardii* (40.9 %) and *Anopheles hyrcanus* (34.1 %), followed by *Culex pipiens* (*sensu lato*) (*s.l*.)/*Cx. torrentium* (7.7 %), *Aedes caspius* (5.7 %), *Cx. modestus* (4.0 %), *An. maculipennis* (*s.l*.) (3.9 %), and *Ae. vexans* (3.0 %). A further seven species were less common in the area studied, including two new records for Romania: *An. algeriensis* and *Ae. hungaricus*. Phylogenetic analysis of COI gene demonstrated the evolutionary relatedness of most species with specimens of the same species collected in other European regions, except *Ae. detritus* and *An. algeriensis*, which exhibited high genetic diversity. Due to the dominance of *Cq. richiardii* and *An. hyrcanus* (75 % of all collected specimens), the overall phenology and temporal pattern of functional groups basically followed the phenology of both species. A huge proportion of the mosquito population in the course of the entire sampling period can be classified as potential WNV vectors. With 40 % of all collected specimens, the most frequent species *Cq. richiardii* is probably the most important vector of WNV in the DDBR.

**Conclusion:**

This is the first DNA-barcoding supported analysis of the mosquito fauna in the DDBR. The detection of two new species highlights the lack of knowledge about the mosquito fauna in Romania and in the DDBR in particular. The results provide detailed insights into the spatial-temporal mosquito species composition, which might lead to a better understanding of mobovirus activity in Romania and thus, can be used for the development of vector control programs.

## Background

In Europe, at least ten different mosquito-borne viruses (moboviruses) are circulating [1] and especially members of the family Flaviviridae, i.e. dengue virus, West Nile virus (WNV), and Usutu virus (USUV), are of growing public health and veterinary importance [2]. Although mosquito and pathogen surveillance in Romania is not performed on a regular basis, the presence of several moboviruses is well known (e.g. WNV, Sindbis virus, Tahyna virus, Lednice virus) [1]. Since the first large WNV outbreak in 1996, with several hundred human cases in Southern Romania [3], WNV has a high relevance for the country. In 2010, another WNV epidemic with more than 50 human cases demonstrated that the virus is widely distributed and established in the country [4].

The Danube Delta is situated in eastern Romania and was formed by Europe’s second largest river discharging into the Black Sea [5]. Under protection since 1991, the Danube Delta Biosphere Reserve (DDBR) covers 580,000 hectares in Romania and 4600 hectares in the Ukraine. The biological diversity in the DDBR is huge, comprising over 1800 species of flora and 3500 species of fauna [6]. Located halfway between the Equator and North Pole, the DDBR is an important hub for migratory birds from Africa and Asia. These circumstances strongly suggest a high risk of introduction of bird associated zoonotic pathogens such as WNV or USUV.

Pathogens imported by migratory birds find a diverse mosquito fauna, which have excellent breeding habitats in this ecologically heterogeneous wetland. Covering more than 30 different ecosystems [5], the DDBR is characterized by vast natural marshes and fresh water bodies, mainly lakes and channels, providing excellent conditions for a diverse and very abundant mosquito fauna [7]. The checklist of the mosquitoes in the DDBR consist of 31 species [7], compromising 56.4 % of the 55 species known for Romania [8–12].

However, regular mosquito monitoring programmes are missing in Romania. As already highlighted by Prioteasa & Falcuta [7], in the DDBR, these types of studies are predominantly hampered by transportation problems, as many areas can only be reached by boat. However, a detailed knowledge on the species composition and phenology are crucially needed to evaluate the risk of pathogen transmission, plan vector control programmes, and to understand the ecology of circulating moboviruses. Therefore, this longitudinal mosquito surveillance study in the DDBR was conducted in order to evaluate the spatio-temporal pattern of the (i) mosquito species composition and diversity, (ii) functional groups of mosquitoes (oviposition sites, overwintering stage, and number of generations), and (iii) the occurrence of potential WNV vectors.

## Methods

### Study area and mosquito sampling

**Fig. 1:**
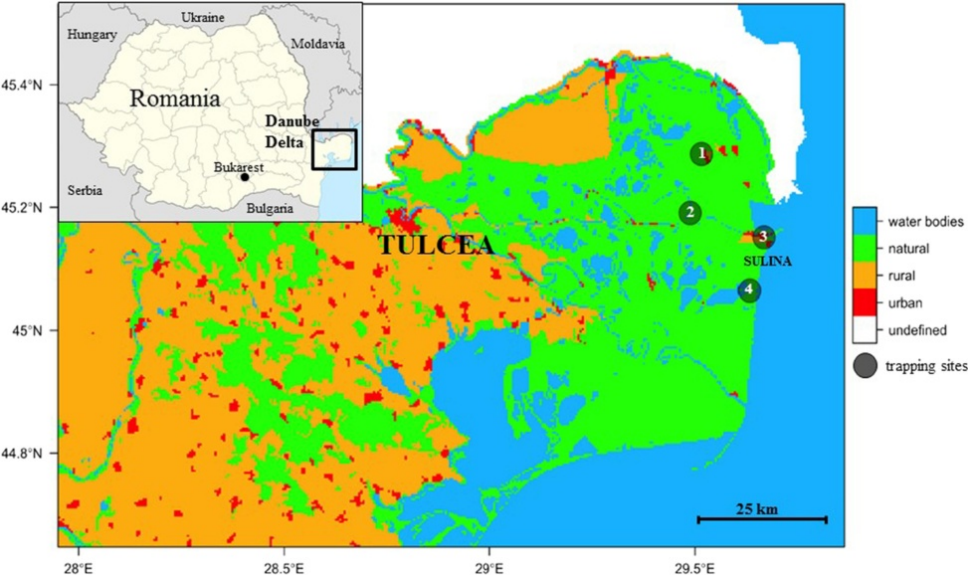
Sampling sites (1: Letea, 2: Dunărea Veche, 3: Sulina, 4: Lake Roșuleț), of mosquitoes in the Danube Delta Biosphere Reserve (Romania) during the sampling period in 2014. Landcover variables are aggregated land cover data [Corine Land Cover (CLC) 2006 raster data, http://www.eea.europa.eu]. CLC-codes: water bodies = 511-523, natural = 311-423, rural = 211-244, urban = 111-142

**Fig. 2:**
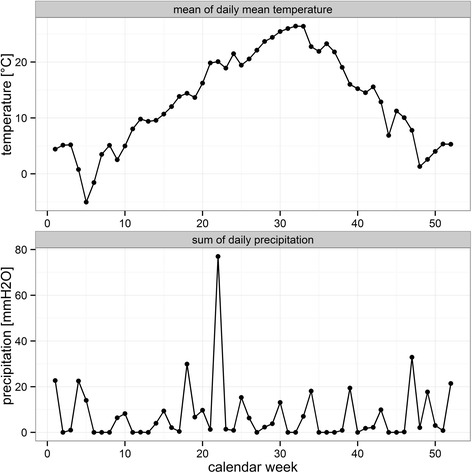
Climate data (mean of the daily mean temperature [°C] and sum of the daily precipitation [mm] per calendar week) for the Danube Delta Biosphere Reserve (Romania) for 2014 downloaded from http://www.ecad.eu/ [41]

Four mosquito trapping sites were selected in the DDBR within an area of about 160 km^2^ and a minimal linear distance of ten kilometres between the sites (Fig. 1). Research permits and approval (9/25.04.2014; 10692/ARBDD/25.04.2014) were issued by the Danube Delta Biosphere Reserve Authority. Between April and September 2014, four carbon dioxide-baited Heavy Duty Encephalitis Vector Survey (EVS) traps (Bioquip Products Inc., California, USA) were operated at each site for one night every tenth day on average. The annual mean temperature of the area is 11 °C (-1 °C in January and 22 °C in July), with a mean precipitation about 350 mm per year (see Fig. 2 for weather conditions during the sampling year 2014).

Trapping site Letea is situated between a channel and a swamp. The biotope is characterized by a few black locusts (*Robinia pseudoacacia*) and mulberry trees (*Morus nigra*) between a small field covered with grasses and a swampy area with reed (*Phragmites australis*) and bulrush (*Typha angustifolia*). Trapping site Dunărea Veche lies on an old, natural branch of the Danube. The high spring water levels flood the area until mid-June connecting the channel with swamps around it in many places. The channel flows very slowly and the soil is permanently moist. Vegetation is dominated by *T. angustifolia*, *P. australis*, *Urtica dioica* and *Fraxinus pallisae*. The trapping site Sulina lies in a black locust tree grove (*R. pseudoacacia*) between a private garden and a stagnant waterbody. The surrounding flora also consists of vines (*Vitis vinifera*) and various species of ruderal herbaceous species. The trapping site Lacul Roșuleț is a platform surrounded by stagnant or very slow flowing water. *T. angustifolia*, *P. australis* and *Salix alba* dominate the surrounding area, which is bordered by big trees which stand up in the flat landscape of vast marshes and lakes.

### Morphological and molecular identification of mosquitoes

Collected mosquitoes were stored, transported to the laboratory on dry ice and morphologically identified on chill tables [13, 14]. Due to transportation or storage, some specimens were damaged and missed relevant characters for the species identification. These were only identified to the genus level or classified as “unidentified”. Selected specimens of all collected species were double-checked by another person without knowing previous identification results. The morphological identification of these specimens was confirmed by the analysis of the mitochondrial cytochrome *c* oxidase subunit I (COI) gene [15]. Mosquitoes were placed in sterile 2 ml reaction tubes and 1.5 ml of cell culture medium (high-glucose Dulbecco’s modified Eagle’s medium [Sigma-Aldrich, St. Louis, MO] with 10 % heat-inactivated foetal bovine serum, 100 U/ml penicillin, 100 μg/ml streptomycin, and 2.5 μg/ml amphotericin B) and 0.75 μl Zirconia beads (Biospec; 2.0 mm beads) were added for homogenization in a TissueLyser (Qiagen, Hilden, Germany) for 2 min at 50 oscillation/s. The suspensions were clarified by centrifugation (5000 g for 1 min), and the supernatant was used for DNA extraction with the RTP Pathogen Kit (Stratec Biomedical AG, Birkenfeld, Germany) according to the manufacturer’s instructions. The extracted DNA of each sample was used as a template for the amplification of ~ 560 bp fragment of the COI gene using the C1-N-2191:5'-GGTAAAATTAAAATATAAACTTC-3'/C1-J-1632:5'-TGATCAAATTTATAAT-3' primers [15]. Each PCR reaction was performed with the HotStartTaq Plus Master Mix Kit (Qiagen, Hilden, Germany) according the manufacturer’s protocol. PCR products were sequenced at least twice in each direction by conventional Sanger technology (LGC, Berlin, Germany).

### Genetic diversity and phylogenetic analysis

Sequence assembly, analysis, and multiple alignments were performed using Geneious v7.1.8 (Biomatters, Auckland, New Zealand). The species-level identification based on COI was conducted with BOLD (http://www.boldsystems.org) and BLAST (http://blast.ncbi.nlm.nih.gov/Blast.cgi). In order to investigate the evolutionary relationship of the mosquito species collected during this study with those previously reported worldwide and available in GenBank, a maximum likelihood (ML) analysis was performed using PhyML 3.0 (http://www.atgc-montpellier.fr/phyml/versions.php) with 1000 pseudoreplicates. To assess the robustness of ML phylogenetic groupings, a bootstrap resampling analysis was conducted using 1000 replicate neighbor-joining (NJ) tree and Kimura-2 distance model in MEGA6 [16]. The Akaike information criterion was chosen as the model selection criterion and the general time-reversible model of sequence evolution with gamma distributed rate variation among sites and a proportion of invariable sites (GTR + I + Γ) as the best model. Sequences were deposited in the GenBank database with the accession numbers KU214640–KU214675 and KT876464–KT876495.

### Data analysis

All other data analysis was conducted with R [17]. The packages plyr [18] and lubridate [19] were used for data manipulation and the packages ggplot2 [20] and gridExtra [21] for data visualization. Due to small variations of the sampling intervals per trapping site, the data were summarized per calendar week. Taxa information on functional characteristics (overwintering stage, oviposition sites, number of generations) and the classification as potential WNV vectors based on the feeding preference were extracted from the literature (Tables 1 and 2). Abundance-based Coverage Estimator (ACE) and Chao1 were used to determine sampling efficiency of mosquito taxa [22–24]. This procedure was performed with the function “EstimateR” from the R package vegan [25].

**Table 1:** Mosquito taxa recorded in the study area of the Danube Delta Biosphere Reserve (DDBR) in Romania during the sampling period in 2014 with the number of specimens collected, their respective overall proportion, information if the species was previously known from Romania and the DDBR, and three functional characteristics for each taxon

Taxa	Specimens (percentage)	Previously known for Romania and the DDBR	Oviposition sites	Overwintering stage	No. of generations	Source for functional classification
*Coquilettidia richiardii* (Ficalbi, 1889)	98276 (40.8552 %)	yes	water	larvae	univoltine	[42]
*Anopheles hyrcanus* (Pallas, 1771)	82073 (34.1193 %)	yes	water	females	multivoltine	[43]
*Culex pipiens* Linnaeus, 1758 (*s.l*.)/Cx. *torrentium* (Martini, 1925)^a^	18416 (7.6559 %)	yes	water	females	multivoltine	[42]
*Aedes caspius* (Pallas, 1771)	13709 (5.6991 %)	yes	land	eggs	multivoltine	[42]
*Culex modestus* Ficalbi, 1890	9534 (3.9635 %)	yes	water	females	multivoltine	[43]
*Anopheles maculipennis* Meigen, 1818 (*s.l*.)^b^	9380 (3.8994 %)	yes	water	females	multivoltine	[42]
*Aedes vexans* (Meigen, 1830)	7295 (3.0327 %)	yes	land	eggs	multivoltine	[42]
Unidentified	1041 (0.4328 %)	-	-	-	-	-
*Anopheles algeriensis* Theobald, 1903	697 (0.2898 %)	no	water	larvae	multivoltine	[43]
*Aedes* sp.	71 (0.0295 %)	-	-	-	-	-
*Aedes detritus* (Haliday, 1833)	31 (0.0129 %)	yes	land	eggs	multivoltine	[42]
*Culex* sp.	10 (0.0042 %)	-	-	-	-	-
*Aedes flavescens* (Müller, 1764)	5 (0.0021 %)	yes	land	eggs	univoltine	[42]
*Aedes hungaricus* Mihályi, 1955	3 (0.0012 %)	no	land	-	-	[14]
*Aedes cinereus* Meigen, 1818	2 (0.0008 %)	yes	land	eggs	multivoltine	[42]
*Culiseta annulata* (Schrank, 1776)	1 (0.0004 %)	yes	water	females	multivoltine	[42]
*Uranotaenia unguiculata* Edwards, 1913	1 (0.0004 %)	yes	water	females	multivoltine	[43]

^a^Selected specimens were identified as *Culex pipiens* Linnaeus, 1758 (*s.l*.) and *Culex pipiens pipiens* Linnaeus, 1758 by DNA-barcoding (Fig. 5), ^b^selected specimens were identified as *Anopheles messeae* Falleroni, 1926 by DNA-barcoding (Fig. 5).

**Table 2:** Mosquito taxa recorded in the study area of the Danube Delta Biosphere Reserve (Romania) during the sampling period in 2014 and the host preference determining the possibility to be a potential bridge vector of West Nile virus

Taxa	Involved in West Nile virus transmission elsewhere	Ornithophilic (bird-biting)	Anthropophilic (human-biting)	Potential bridge vector (readily bites both birds and humans)	Source for classification
*Coquilettidia richiardii* (Ficalbi, 1889)	yes	yes	yes	yes	[44]
*Anopheles hyrcanus* (Pallas, 1771)	yes	no	yes	no	[14]
*Culex pipiens* Linnaeus, 1758 (*s.l.*) /Cx. *torrentium* (Martini, 1925)^a^	(yes)^c^	(yes)^c^	(yes)^c^	yes	[44]
*Aedes caspius* (Pallas, 1771)	yes	no	yes	no	[44]
*Culex modestus* Ficalbi, 1890	yes	yes	yes	yes	[44]
*Anopheles maculipennis* Meigen, 1818 (*s.l*.)^b^	yes	no	yes	no	[44]
*Aedes vexans* (Meigen, 1830)	yes	no	yes	no	[44]
Unidentified	-	-	-	unclassified	-
*Anopheles algeriensis* Theobald, 1903	no	no	yes	no	[44]
*Aedes* sp.	-	-	-	unclassified	-
*Aedes detritus* (Haliday, 1833)	no	yes	yes	yes	[44]
*Culex* sp.	-	-	-	unclassified	-
*Aedes flavescens* (Müller, 1764)	no	no	yes	no	[44]
*Aedes hungaricus* Mihályi, 1955	no	no	yes	no	[14]
*Aedes cinereus* Meigen, 1818	yes	yes	yes	yes	[44]
*Culiseta annulata* (Schrank, 1776)	no	yes	yes	yes	[44]
*Uranotaenia unguiculata* Edwards, 1913	yes	no	no	no	[14, 45]

^a^Selected specimens were identified as *Culex pipiens* Linnaeus, 1758 (*s.l*.) and *Culex pipiens pipiens Linnaeus*, 1758 by DNA-barcoding (Fig. 5), ^b^selected specimens were identified as *Anopheles messeae* Falleroni, 1926 by DNA-barcoding (Fig. 5), ^c^ *Culex pipiens* (*s.l*.) and Cx. *torrentium* were not differentiated for most of the collected specimens

## Results

### Mosquito species composition

A total of 240,546 female mosquito specimens belonging to 8 genera and 14 taxa were successfully identified by morphological characteristics (Tables 1 and 2). The seven dominant taxa, with more than 2000 individuals each, were *Coquilettidia richiardii* (40.9 %), *Anopheles hyrcanus* (34.1 %), *Culex pipiens* (*sensu lato*) (*s.l*.)/*Cx. torrentium* (7.7 %), *Aedes caspius* (5.7 %), *Cx. modestus* (4.0 %), *An. maculipennis* (*s.l*.) (3.9 %), and *Ae. vexans* (3.0 %). Among the rare species, representing 0.7 % of all collected individuals, were *Ae. detritus, Ae. flavescens*, *Ae. cinereus*, *Culiseta annulata*, and *Uranotaenia unguiculata*. In addition, we detected two new species for Romania: *An. algeriensis* and *Ae. hungaricus*, which both have been morphologically and genetically confirmed.

**Fig. 3:**
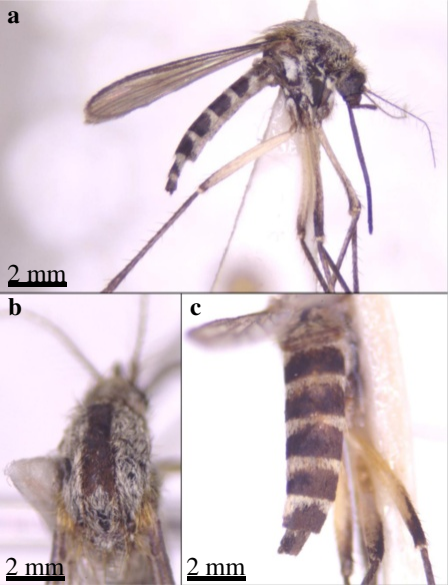
Specimen of *Aedes hungaricus* detected in the Danube Delta Biosphere Reserve (Romania) during the sampling period in 2014. **a** Lateral view; **b** Scutum; **c** Abdomen

In Letea, three females of *Ae. hungaricus* were trapped between 29^th^ June and 9^th^ July 2014. These were identified according to the following morphological characteristics [14, 26]: small species, with blackish brown scaled proboscis and palps, occiput with narrow whitish scales dorsally, broad whitish scales and scattered dark scales laterally, scutum covered with greyish white scales and a median stripe of dark brown scales, scutellum with pale narrow scales, hypostigmal scale patch absent, upper mesepisternal scale patch reaches the anterior angle of the mesepisternum, mesepimeral scale patch does not reach the lower margin of the mesepimeron, femora of the fore legs predominately pale scaled in the basal half, tibiae of the hind legs with dark scales on the anterior surface, tarsomeres dark scaled without pale basal rings, wing veins covered with dark scales, abdominal terga with blackish brown scales and pale basal bands, which are slightly narrower in the middle and connected with pale lateral triangular patches (Fig. 3). Not all characteristics could clearly be seen on each specimen, because of damage due to transportation and storage. Therefore, a reference adult female collected as larva in 1998 on the Tisa river close to Mártély in Hungary was taken for morphological comparison. The specimen from Hungary was independently identified as *Ae. hungaricus* from three entomologists and the overall appearance was in agreement with the three specimens from Romania.

**Fig. 4:**
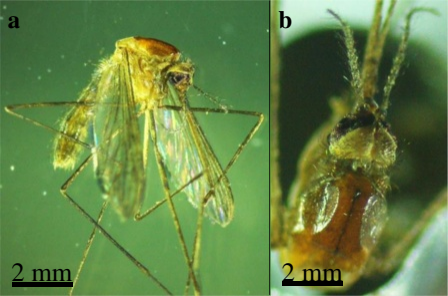
Specimen of *Anopheles algeriensis* detected in the Danube Delta Biosphere Reserve (Romania) during the sampling period in 2014. **a** Lateral view; **b** Scutum and head

A second new species, *An. algeriensis*, was found with 697 females (0.3 % of all collected mosquito specimens) at all four sampling sites between April and September 2014. Typical morphological characteristics have been observed [14]: head antennal ornamentation rare and poorly developed whorls without a tuft or long white scales on interocular apse, maxillary palpus is entirely dark, no white rings, thorax covering of scutum with setae only, hind leg colour of tarsomeres entirely dark and mostly with a small apical ring, wings ornamentation entirely dark and without spot on the costal margin (Fig. 4).

All mosquito specimens were homogenized for further pathogen screening. Extracted DNA is stored in the Bernhard Nocht Institute for Tropical Medicine, WHO Collaborating Centre for Arbovirus and Haemorrhagic Fever Reference and Research National Reference Centre for Tropical Infectious Diseases, Hamburg, Germany.

### DNA barcoding and phylogeny of mosquito species

**Fig. 5:**
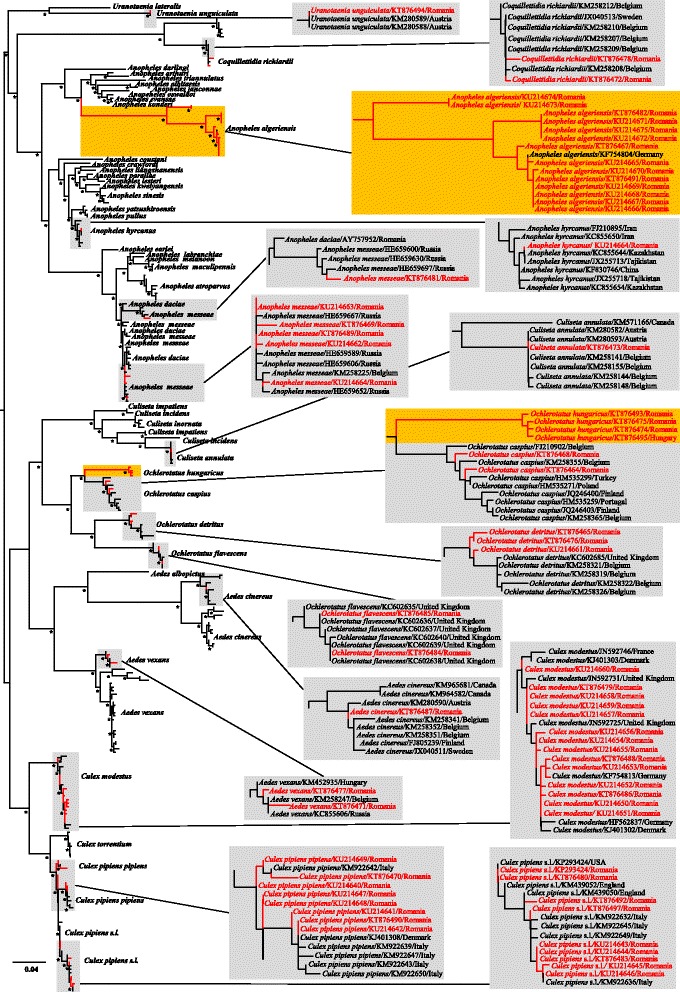
Maximum-likelihood phylogeny of the COI gene sequences for selected specimens of the 14 collected mosquito species detected in the Danube Delta Biosphere Reserve (Romania) during the sampling period in 2014 (red font) and additional sequences retrieved from the NCBI nucleotide database (http://www.ncbi.nlm.nih.gov). Red marked sections of the tree and the magnified areas in grey/orange indicate the location of the mosquito species detected in this study. The clades including *Aedes hungaricus* and *Anopheles algeriensis* (first reports for Romania) are highlighted in orange. The maximum likelihood bootstrap replicates (≥70 %) and parallel NJ bootstrap values above 70 (1000 replicates) are indicated with an asterisk at the nodes. The scale-bar indicates the genetic distance scale expressed as mean number of nucleotide substitutions per site

COI sequences of ~ 550 bp were successfully amplified from 66 mosquito specimens from the DDBR and compared with those currently available in databases. Four sequences of *Ae. hungaricus* are submitted as the first records for public databases. The alignment was unambiguous without gaps and stop codons in amino acid translation. Comparisons of the COI sequence alignment indicated point mutations for all detected mosquito species with the highest number observed in *An. algeriensis* (number of point mutations [npms] = 40), followed by *Cx. pipiens* (*s.l*.) (npms = 15) and *Ae. detritus* (npms = 13). No deletion or insertion among the sequenced samples have been observed. Gene sequences of *Ae. cinereus* (*n* = 1), *Ae. vexans* (*n* = 2), *An. hyrcanus* (*n* = 1), *An. messeae* (morphologically identified as *An. maculipennis* (*s.l*.) (*n* = 5), *Cs. annulata* (*n* = 1), *Cq. richiardii* (*n* = 2), *Cx. pipiens pipiens* (n = 8), *Cx. pipiens* (*s.l.*) (morphologically identified as *Culex pipiens* (*s.l.*)/*Cx. torrentium*) (*n* = 9), *Cx. modestus* (*n* = 14)*, Ae. flavescens* (*n* = 2), *Ae. caspius* (*n* = 2), and *Ur. unguiculata* (*n* = 1) from the DDBR were very similar to sequences obtained from mosquitoes collected in other European regions, except for *An. algeriensis* (*n* = 14) and *Ae. detritus* (*n* = 3), which exhibited relatively high intraspecific divergence (6 and 3 %, respectively). These results are supported by the phylogenetic analysis, which demonstrated the close evolutionary relatedness and a similar clustering of the above mentioned species with specimens of the same taxon from other regions (Fig. 5). Due to missing COI or other gene sequences of *Ae. hungaricus* in the databases, the phylogenetic clustering of this particular species should be interpreted with caution. However, the analysed specimens of this species formed a distinct and highly supported monophyletic clade, which is clustered with *Ae. caspius* in a distinct group within the *Aedes* phylogeny (Fig. 5). It is important to note that the sequences of the *Ae. hungaricus* specimens from DDBR and the reference specimen from Hungary were almost identical. *Anopheles algeriensis* forms a highly divergent paraphyletic group with several lineages (likely new subspecies) within the genus *Anopheles*. The overall clustering pattern of the phylogenetic tree was similar to that of NJ tree (data not shown), and all species branched with their respective subfamilies.

### Data analysis

Between eight and twelve taxa were recorded at the four sampling sites (Table 3). Except for the sampling site Sulina, with 12 observed and 15 estimated taxa (20 % difference), the ACE and Chao1 indices estimated the same number of taxa as observed, suggesting a good coverage of the taxa present in the study area.

**Table 3:** Estimated taxa richness according the abundance-based coverage estimator (ACE) and Chao1 for the four study sites in the Danube Delta Biosphere Reserve (Romania) during the sampling period in 2014

	Dunărea Veche	Lacul Roșuleț	Letea	Sulina
Observed number of taxa	8.000	9.000	12.000	12.000
Chao1	8.000	9.000	12.000	15.000
Chao1 standard error	0.000	0.000	0.000	4.517
ACE	8.000	NaN^a^	13.380	NaN^a^
ACE standard error	0.935	NaN^a^	1.708	NaN^a^

^a^Calculation of the ACE not possible, because all rare species (<10 specimens) contained only a single specimen

**Fig. 6:**
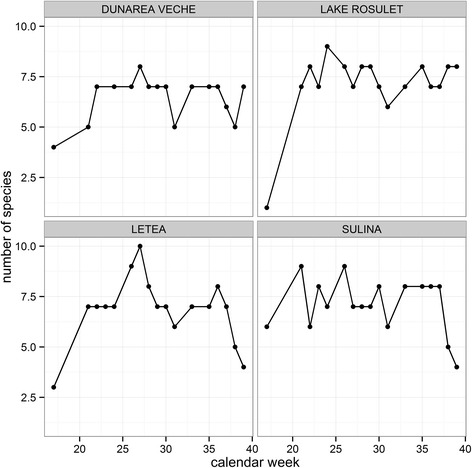
Number of detected mosquito taxa per calendar week for the four sampling sites in the Danube Delta Biosphere Reserve (Romania) during the sampling period in 2014

**Fig. 7:**
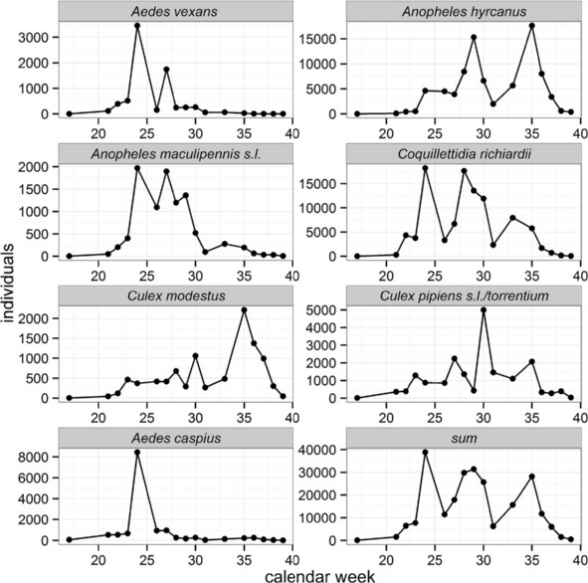
Number of detected specimens per calendar week of the seven most common mosquito taxa (>2000 specimens) detected in the Danube Delta Biosphere Reserve (Romania) during the sampling period in 2014

The number of detected taxa per sampling site and calendar week varied from three to ten with the lowest taxa richness for the first sampling in April and highest number of detected taxa in June (Fig. 6). The highest numbers of mosquito specimens per calendar week were collected at the beginning of June, followed by two peaks at the end of June and August. The detected taxa showed different phenological patterns (Fig. 7). For example, the highest number of specimens for *Ae. vexans* and *Ae. caspius* were trapped early in the year, whereas most *Cx. modestus* were sampled in the late summer. Another example is the number of observed generations, e.g. *Ae. caspius* showed a single population peak, while *Cq. richiardii* and *An. hyrcanus* had three and two population peaks, respectively (Fig. 7).

**Fig. 8:**
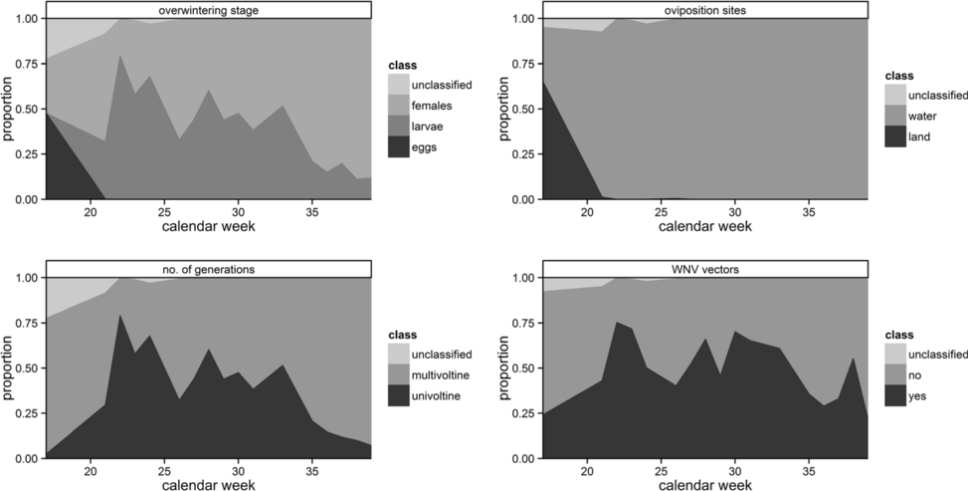
Proportion of three functional groups and West Nile virus vectors of the total catch of mosquitoes per calendar week in the Danube Delta Biosphere Reserve (Romania) during the sampling period in 2014

The mosquito population over the sampling period was dominated by taxa, which lay their eggs on the water surface, whereas taxa laying their eggs on the soil were only present at the beginning of the sampling period (Fig. 8). The overwintering stages of the taxa followed a series with the highest proportion of species overwintering in the egg stage at the beginning of the year, followed by taxa overwintering in the larval stage, and were finally dominated by taxa, which overwinter as females. Univoltine taxa had their highest proportion during the summer months, whereas multivoltine taxa were present during the entire sampling period. Potential WNV vectors were also present during the entire sampling period, accounting for more than 50 % of the total number of collected specimens and exceeding 50 % of all collected specimens for most calendar weeks in the summer.

## Discussion

This study confirmed 12 previously recognized mosquito species for Romania by combining morphological identification and sequencing of the COI gene, representing one fifth (21.8 %) of the known 55 species of the country [8–12]. Both indices for extrapolated taxa richness, bias-corrected Chao and ACE, indicated a relatively good coverage of the mosquito taxa collected with EVS traps for the studied area. However, at the same time, the first reports of two mosquito species for Romania, *An. algeriensis* and *Ae. hungaricus*, highlight the lack of knowledge about the mosquito fauna of the country and the DDBR in particular. These new records were demonstrated, because a huge number of specimens of nearly one quarter of a million mosquitoes was collected over the entire vegetation period. Furthermore, the sampling sites included remote areas of the DDBR only accessible by boat. Only three specimens of *Ae. hungaricus* were found. Due to its general rarity in Europe, the ecology of this species is largely unknown [14]. It was only described that the larvae develop in floodwater pools in river valleys and probably have several generations per year [27]. With 697 specimens, *An. algeriensis* was trapped more frequently, but representing only 0.3 % of all collected mosquito specimens. The species is widespread in Europe with a distribution centre in the Mediterranean region, but was also found in central Europe as far to the north in England or Germany [14, 28–30]. Larval breeding sites are generally located in marshes and slow running brooks covered with dense vegetation [14], which are also present in the study area. Females of the species bite mammals outside, near their breeding sites and are susceptible to *Plasmodium* spp. [14]. However, due to their scarcity, both new species probably do not play an important role as vectors of pathogens in Romania [30].

In order to avoid incorrect mosquito species identification, selected specimens of each morphological identified mosquito species were used for a DNA-barcoding approach. The analysis of the intraspecific sequence variation (6 %) of the *An. algeriensis* COI gene revealed the existence of at least three new relatives. This result is supported by the phylogenetic analysis suggesting the occurrence of a heterogeneous *An. algeriensis* population within a relatively small region. Such differentiation might be especially important if the different subpopulations have a different vector competence [31]. The congruence between morphology-based identification and DNA-barcode grouping based on phylogenetic clustering with high bootstrap support (≥95 %) was found for all morphologically identified taxa. Therefore, morphology-based identification is appropriate to identify the mosquito species in the study area. However, especially the detection of cryptic species (e.g. *Culex pipiens* (*s.l*.)/*Cx. torrentium* or the members of the *Anopheles maculipennis* complex) probably require a mass screening *via* specific PCRs [32], [33] rather than a DNA-barcoding approach.

The main difficulty in the phylogenetic tree reconstruction was the unbalanced amount of available nucleotide sequences from other countries. However, the mitochondrial gene (COI) based phylogeny clearly related the DDBR mosquito species to those collected in other European countries and provided evidence for population subdivision in *An. algeriensis* and *Ae. detritus*. Such differences suggest allopatric speciation evolvement or mixing of different mosquito populations, which developed in distinct geographic regions. Another interesting point worth mentioning here, is the phylogenetic clustering of *Ae. hungaricus*. Although the latter seems to be a homogenous species, almost identical with the reference specimen from Hungary, further studies on genetic diversity of this rare species from other countries are necessary for a final assessment.

The mosquito fauna of the trapping sites was clearly dominated by two species: *Cq. richiardii* and *An. hyrcanus*. For Romania, both species were previously reported to have their main distribution in the DDBR and surrounding floodplains [10]. *Coquillettidia richiardii* has a specialized life-cycle with larvae and pupae living permanently submerged and obtaining oxygen from the aerenchyma of various aquatic plants in permanent water bodies, finding perfect conditions in the DDBR. Similar breeding site preferences for stagnant water bodies with rich aquatic vegetation were described for *An. hyrcanus*. Both species are multivoltine [14, 34] and had two (*An. hyrcanus*) and three populations peaks (*Cq. richiardii*) during the study year. Due to their dominance, representing over three quarters of all collected specimens, the overall phenology and temporal pattern of functional groups basically followed the pattern of both species with three distinct population peaks, domination of the oviposition site “water” (both species) and domination of the overwintering stage “larvae” (*Cq. richiardii*) and “female” (*An. hyrcanus*).

Between 2011 and 2013, different mosquito species in Romania have been tested WNV-positive [35]. *Culex pipiens* (*s.l*.) is considered to be the most important WNV vector in the country [35, 36], and together with *Cx. modestus* considered to be the main vector species of WNV in Europe [37, 38]. However, in Romania, WNV was also detected in mosquito pools of the species *Cq. richiardii*, *An. hyrcanus*, *Ur. unguiculata*, *Ae. caspius*, and *An. maculipennis* (*s.l*.). Nicolescu [36] highlighted that these species might play an important role in the transmission cycle of WNV, if the principal vector species are missing or present only with low densities. During the entire sampling period, a huge proportion of the mosquito population can be classified as potential WNV vectors. With 40 % of all collected specimens, the most frequent species *Cq. richiardii* is probably the most important vector of WNV in the DDBR, followed by *Cx. pipiens* (*s.l*)/*Cx. torrentium*, *Ae. caspius* and *Cx. modestus*, which were all found WNV-positive in Romania [35]. *Anopheles hyrcanus* was the second most frequent species and also detected WNV-positive in the country [35, 39]. However, due to the generally assumed host preference for mammals, the species probably does not play an important role as bridge vector.

## Conclusion

The data generated during this study is likely biased, because it only included four sampling sites and one type of adult trap (e.g. different types of adult traps are known to have a different trapping performance) [40]. Therefore, an increase of sampling sites and the use of diverse trapping methods (e.g. different types of adult traps or gravid traps) including the collection of immature stages might allow the detection of more mosquito species. Nevertheless, these data from one vegetation period provide a first, but detailed overview of the mosquito communities in the DDBR. Thereby, the detection of two new mosquito species highlights the lack of knowledge about the composition and genetic diversity of the mosquito fauna in Romania and in the DDBR in particular. The greatest proportion of collected specimens could be classified as potential WNV vectors, which can account for up to 70 % of all sampled mosquitoes per calendar week. The extension of the entomological surveillance programme will provide baseline data, which are necessary to better understand mobovirus activity and the phylogeography of a medically important mosquito vector species. Finally, this information can also help to implement vector control programmes, e.g. to adjust the timing of interventions.

## Abbreviations

ACE: abundance-based coverage estimator
Chao1: estimator of species richness named after the inventor of the index (Chao [27, 28])
COI: cytochrome oxidase I
DDBR: Danube Delta Biosphere Reserve
DNA: deoxyribonucleic acid
EVS: Heavy Duty Encephalitis Vector Survey
mobovirus: mosquito-borne virus
PCR: polymerase chain reaction
RNA: ribonucleic acid
USUV: Usutu virus
WNV: West Nile virus
